# Sensorimotor adaptation in spatial orientation task: a fNIRS study

**DOI:** 10.1038/s41598-023-42416-3

**Published:** 2023-09-13

**Authors:** Sang Seok Yeo, Tae Su Jang, Seong Ho Yun

**Affiliations:** 1 Department of Physical Therapy, College of Health and Welfare Sciences, Dankook University, Cheonan, Chungcheongnam-do Republic of Korea; 2 Department of Health Administration, College of Health and Welfare Sciences, Dankook University, Cheonan, Chungcheongnam-do Republic of Korea; 3 Department of Public Health Sciences, Graduate School, Dankook University, Cheonan, Chungcheongnam-do Republic of Korea

**Keywords:** Neuroscience, Neurology

## Abstract

In sensorimotor conflicts, the brain forms and updates a new sensorimotor relationship through sensorimotor integration. As humans adapt to new sensorimotor mapping, goal-directed movements become increasingly precise. Using functional near-infrared spectroscopy, we investigated the changes in cortical activity during sensorimotor adaptation in a spatial orientation task with sensorimotor conflict. Individuals performed a reversed spatial orientation training in which the visual feedback guiding hand movements was reversed. We measured cortical activity and spatial orientation performance, including the response time, completion number, error, and accuracy. The results revealed the continuous activation in the left SMG during sensorimotor adaptation and decreased activation in the right SAC, AG and SMG after sensorimotor adaptation. These findings indicated the contribution of the left SMG to sensorimotor adaptation and the improved efficiency of cortical activity after sensorimotor adaptation, respectively. Our studies suggest the neural mechanisms related to sensorimotor adaptation to a reversed spatial orientation task.

## Introduction

Sensorimotor integration is often disrupted when predicted and perceived sensory feedback conflicts in daily life^1^. As a result, sensorimotor conflict can cause perturbations of motor commands during goal-directed movements. For example, when the direction of the cursor displayed on a computer screen is opposite the movement of the hand, the cursor movement is distorted away from the target while reaching a visual target. During continuous sensorimotor conflict, the sensorimotor system aims to compensate for the perturbations, reduce error, and return to its former performance level. This process is referred to as sensorimotor adaptation, and involves two mechanisms^2^. One is explicit adaptation, which employs a cognition-based motor strategy to reduce error rapidly at the beginning of adaptation^3^. The other is implicit adaptation, a slow and automatic process necessary to develop or update a new sensorimotor mapping^3, 4^. As individuals adapt to a new sensorimotor relationship, goal-directed movements are more accurate, thereby reducing errors. To confirm whether a new sensorimotor mapping has been formed, there is a method to re-expose a situation in which the sensorimotor conflict is removed^5^. When sensorimotor conflict is removed after a new sensorimotor mapping, individuals experience errors and perturbation in the direction opposite to the initial sensorimotor conflict. These errors, which are referred to as after-effects, represent the underlying neural adaptation to sensorimotor mapping^6, 7^.

Previous studies have extensively investigated how the brain integrates sensory input and motor output and adapts to new sensorimotor mapping. In the functional magnetic resonance imaging (fMRI) studies, neural plasticity in the posterior parietal cortex, including somatosensory association cortex (SAC), supramarginal gyrus (SMG), angular gyrus (AG), plays a role in forming and adapting a new sensorimotor mapping in the condition of sensorimotor conflict^5, 8, 9^. Specifically, the SAC is associated with integration of visuospatial, vestibular, somatosensory and motor information^10, 11^. In addition, the primary function of SMG and AG is to remap sensorimotor information and adapt a new sensorimotor mapping based on the error correction and recalibration^12, 13^.

Despite the existing knowledge, it is important to point out that sensorimotor adaptations in previous fMRI studies were conducted in a supine position due to structural limitations. The individua’s posture directly affects sensorimotor integration and adaptation because of altered somatosensory and vestibular input^14–16^. Among functional neuroimaging techniques, functional near-infrared spectroscopy (fNIRS) has the advantage of being less susceptible to motion artifacts and unaffected by measurement posture^17, 18^. fNIRS is a non-invasive optical method that indirectly detects cortical activity based on hemodynamic response and is considered a promising neuroimaging technique for actual sensorimotor tasks. Furthermore, although previous studies have described sensorimotor adaptation using clockwise distorted reaching tasks, sensorimotor adaptation in everyday life is a more complexes process involving various cognitive components and strategic correction^19, 20^. Therefore, it is necessary to determine how the brain responds during sensorimotor adaptation, which requires more complex cognition ability than reaching tasks. The trail making task (TMT) demand several cognitive processes, such as spatial orientation, visuomotor abilities, and cognitive flexibility^21^. Among these functions, spatial orientation refers to the ability to identify the position or direction of objects or points in the surrounding environment. This ability affects the reconstruction of a new mapping between the visual and motor space for sensorimotor adaptation^22^.

In the current study, we examined the changes in cortical activity and spatial orientation performance during sensorimotor adaptation to reversed spatial orientation tasks.

## Methods

### Participants

A total of 10 healthy adults (5 men and 5 women; mean age: 23.3 years; right-handed) were recruited for this study. G-power software (G*power 3.1.9.4, Heinrich-Heine-Universität, Düsseldorf, Germany) was used to calculate the sample size. A minimum of 5 participants was adequate to power the study (Statistical test = ANOVA: Repeated Measures, within factors; Effect size = 0.79; a significance level = 0.05; Power = 0.95; Number of groups = 1; Number of measurements = 5; Corr among rep measures = 0.5; Nonsphericity correction = 1). The effect size was chosen based on findings by Lefarancois and Messier^23^ with respect to visuomotor adaptation in virtual reality environment. None of the participants had a history of musculoskeletal, neurological, or psychiatric diseases that could affect their performance on the spatial orientation task. All experiments were conducted in accordance with relevant guidelines and regulations from Declaration of Helsinki. This study protocol was approved by the Institutional Review Board of the Dankook University (DKU 2023-01-016-001). All participants were given detailed instructions regarding the experiment, provided written informed consent to participate, and compensated $10 per session for their participation.

### Measurements

#### Trail making task (TMT) and reversed trail making task (rTMT)

The TMT is a popular neuropsychological test for the evaluation of spatial orientation, working memory, and executive function^24^. The experiment in the present study had two main tasks, including the TMT and rTMT. During the TMT, the participants were asked to connect the encircled numbers from 1 to 25, which were scattered randomly on a monitor, in an ascending order (1–2–3…). In the rTMT, the cursor was inverted left and right based on the y-axis to cause sensorimotor conflict. For example, left mouse movements displaced the cursor to the right and vice versa. Participants were asked to perform the TMT and rTMT as quickly and precisely as possible. Performance of spatial orientation was evaluated by the response time, completion number, error, and accuracy. The response time is defined as the time required to connect from one number to the nest. The completion number indicates the final connected number during the limited time of 40 s. The errors were recorded when individuals incorrectly sequenced either number (e.g., 1–2–5)^25^. Accuracy was calculated as a percentage of correct trials, which subtracted error trials, out of the completion number.

#### Functional near infrared spectroscopy

Continuous wave fNIRS (NIRSport 2, Nirx Medical Technologies LLC, Berlin, Germany) was used to record hemodynamic responses and cortical activity during TMT and rTMT with a sampling rate of 12.52 Hz. Nirsport 2 is a wearable fNIRS system that is specifically designed to maximize the signal-to-noise ratio in a mobile environment^26^. We employed thirteen light sources and fourteen detectors to record the optical density at two different wavelengths (760 and 850 nm). The light sources and detectors arrangement covered a total of 41 channels for data acquisition. The montage was designed in accordance with international 10–20 systems using NIRSite software (NIRx Medical Technologies, LLC, LA, USA) and the fNIRS Optode’s Location Decider toolbox^27^. The regions of interest were the SAC (BA 5 and 7), primary visual cortex (BA 17), secondary visual cortex (BA 18), third visual cortex (BA 19), AG (BA 39), and SMG (BA 40).

### Procedure

Participants were seated in a comfortable chair in front of a table with a monitor placed 60 cm away from them. In the pre-test, the participants performed two experimental sessions: the TMT and rTMT. Each session consisted of a block paradigm design (three cycle; 40 s resting and 40 tasks). During the resting state, the participants were instructed to fix a cross in the center of a black screen. During the task, the participants were instructed to connect the circles as quickly as possible without lifting the mouse. Participants who completed the task within 40 s were instructed to close their eyes and remain in a stable state. Participants who were not able to complete the task within 40 s were instructed to stop immediately. There was a 5-min rest interval between the two experimental sessions. Then, the participants engaged in the rTMT training, which was carried out for 5 days with 20 sessions per day. On days 3 and 5 of rTMT training, middle and post-tests were conducted to determine the degree of adaptation to sensorimotor conflict. Finally, the participants performed TMT to confirm whether a new sensorimotor mapping was formed. We obtained fNIRS and performance of spatial orientation task data, including response time, completion number, errors, and accuracy during all sessions.

### Data analysis

#### Performance of the spatial orientation task

The performance of spatial orientation task analysis was performed using the SPSS software (version 21.0; SPSS Inc., Chicago, IL, USA). The Shapiro–Wilk test was used to examine the normality of the data. Since response time and accuracy data were not normally distributed, the Friedman test was conducted to analyze the significance of the differences in response time and accuracy between sessions. When significant differences were found, post hoc analysis was subsequently performed using a Wilcoxon’s signed rank test with Bonferroni correction. The repeated measure ANOVA was performed to analyze the significant difference in completion numbers and errors. Post hoc testing was accomplished using paired sample t-test with Bonferroni correction for multiple comparisons. Null hypotheses of no difference were rejected if p-values were < 0.05.

#### fNIRS data

The fNIRS data were processed using NIRSlab (nirsLAB version 2019.04; NIRx Medical Technologies LLC, Berlin, Germany). The raw data were preprocessed by removing the discontinuities and spike artifacts, which is inherent function in NIRSlab^28^. Discontinuties were automatically detected and removed (std threshold = 5)^29^. Then, two independent researchers inspected the fNIRS signal to individuate the spike artifacts to be removed. Spike artifacts were only marked as such, if both researchers agreed. Spike artifacts were replaced with automatically random signals (a set of random numbers that are sampled from a Gaussian distribution, with a standard deviation equal to the average of the 4-s time intervals preceding and following the motion artifacts, and with a mean equal to the data value)^28^. Subsequently, the data were filtered in the band-pass of 0.01–0.20 Hz and a 15% roll off width to eliminate the effects of heartbeat, respiration, and low-frequency signal drifts for each wavelength^28^. The modified Beer-Lambert law was used to convert optical density to oxyhemoglobin (HbO) and deoxy-hemoglobin (HbR) concentrations^30, 31^. We performed baseline correction that defined as the 10 s prior to first task block to accounts for the individual variability^32, 33^. We used HbO, HbR, and total hemoglobin (HbT) data for further analysis.

For topographical analysis, we used the Statistical Parameter Mapping NIRS-SPM (SPM 8) tool executed in the NIRSlab (version 2019.4). The general linear model (GLM) with a canonical hemodynamic response curve, as incorporated in nirsLAB, was used to analyze the significant task-related cortical activation, separately for HbO, HbR, and HbT, for each individual^30^. The GLM was evaluated using the equation, Y = Xβ + E, where Y is the matrix of hemodynamic data; X is the n x p design matrix (n is the number of rows and p the number of columns of the design matrix); β is the GLM-coefficient matrix and E is the residual term. SPM-1 within subject analysis was performed to estimate the degree of activation in each channel with respect to the basline^34^. In SPM-1 analysis, a canonical haemodynamic response function (HRF) was considered and pre-whitening was omitted. This was followed with the application of a Discrete Cosine Transformation (DCT) temporal parameter with a highpass period cut-off of 128 s. A Gaussian Full Width at Half Maximum (FWHM) 4 model was applied, and for each participant General Linear Models (GLMs) were obtained based on the HbO and HbT signals. The design matrix was set up to contrast the rest (0) / task (1)^35^. For group analysis of the brain activity in accordance with the statistical analysis, SPM-2 between subject analysis was used. SPM-1 and SPM-2 t-mpas were conducted based on those t-contrasts with *p* < 0.05 (uncorrected)^36^. The beta-coefficient of HbO, HbR, and HbT for each sessions was extracted from the GLM of each participant to evaluate the difference in cortical activity between sessions. The beta-coefficient, representing the amplitudes of the hemodynamic responses, indicates the intensity of cortical activation^37^. The Friedman test was performed for each channel to analyze the significant differences in the beta coefficient of HbO, HbR, and HbT between sessions. The p-values were corrected by FDR for multiple comparisons of the channels. In the significant channels, post hoc analysis was performed using a Wilcoxon’s signed rank test with FDR. This study focused on two comparisons to identify the cortical activity associated with sensorimotor adaptation, and after-effects; pre-rTMT vs post-rTMT (sensorimotor adaptation); pre-TMT vs post-TMT (after-effects).

## Results

### Performance of spatial orientation task

**Figure 1:**
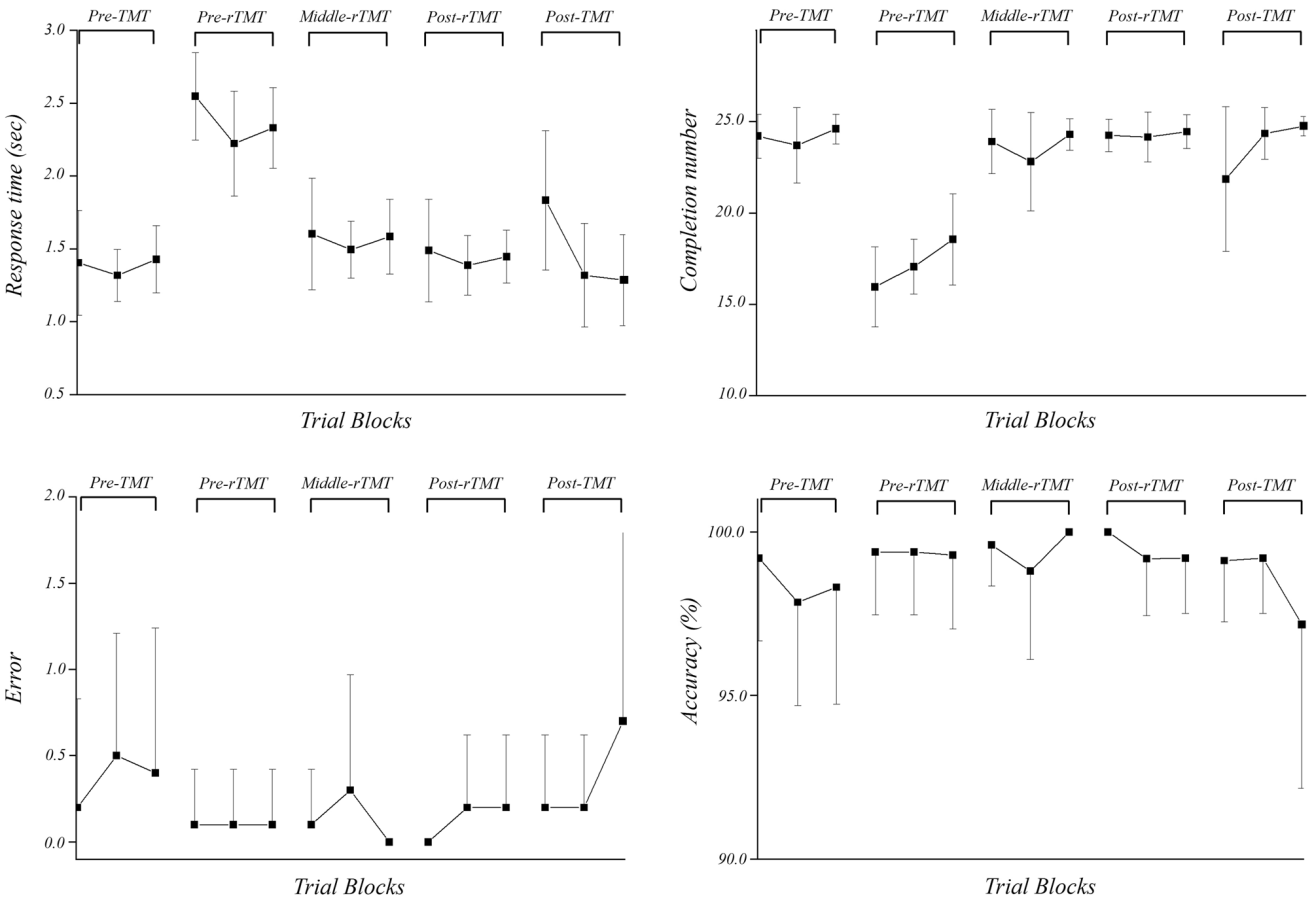
The performance of spatial orientation task over sessions. The square indicates the mean values in each block. TMT, trail making task; rTMT, reversed trail making task.

**Figure 2:**
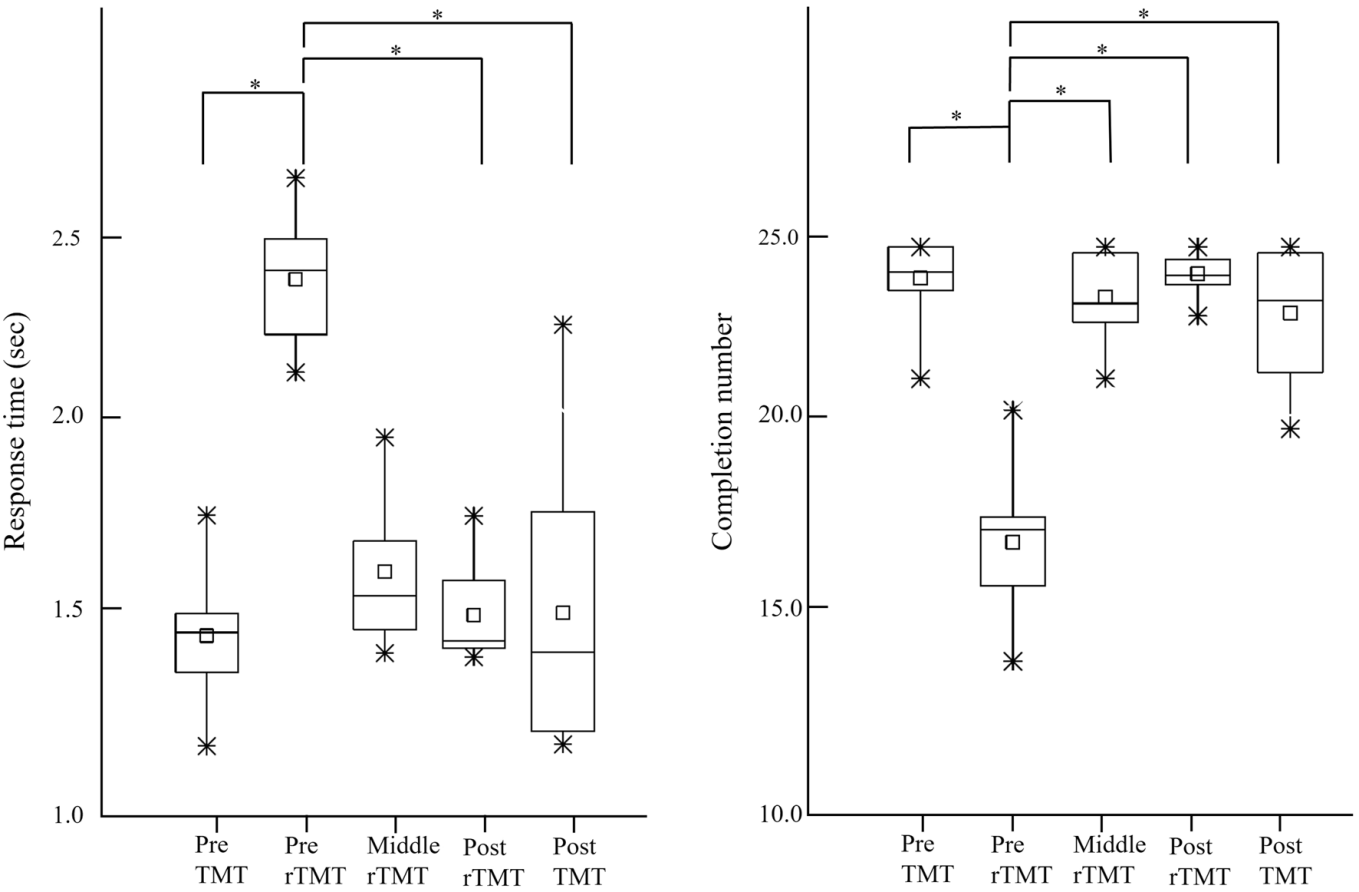
The post hoc analysis in response time. Data are presented as mean values ± SD. TMT, trail making task; rTMT, reversed trail making task. *Indicates statistical differences as confirmed by Wilcoxon’s signed-rank or paired sample t-test with Bonferroni correction (*p*_corrected_ < 0.05).

Figure 1 showed performance of spatial orientation task during each session. There were significant differences in response time (*p* = 0.001) and completion number (*p* < 0.001) between the sessions (Table 1). The post hoc analysis revealed that the response time during pre-rTMT was longer than that during pre-TMT (*p*_*corrected*_ < 0.001), post-rTMT (*p*_corrected_ = 0.002), and post-TMT (*p*_*corrected*_ = 0.001) (Fig. 2). The completion number of pre-rTMT was lower than pre-TMT (*p*_*corrected*_ < 0.001), middle-rTMT (*p*_*corrected*_ = 0.030), post-rTMT (*p*_*corrected*_ = 0.001), and post-TMT (*p*_*corrected*_ = 0.030) (Fig. 2). There was no significant difference in error and accuracy between the sessions (*p* > 0.05) (Table 1).

**Table 1:** The performance of trail making task and reversed trail making task.

	Pre-TMT	Pre-rTMT	Middle- rTMT	Post-rTMT	Post-TMT	*p*
Response time (ms)	1.38 ± 0.17	2.37 ± 0.18	1.56 ± 0.21	1.44 ± 0.13	1.45 ± 0.38	.001*
Completion number	24.17 ± 1.08	17.17 ± 1.77	23.67 ± 1.13	24.28 ± 0.58	23.25 ± 1.75	< 0.001*
Error	0.37 ± 0.48	0.10 ± 0.16	0.13 ± 0.23	0.13 ± 0.23	0.36 ± 0.57	.169
Accuracy (%)	98.45 ± 2.02	99.36 ± 1.04	99.47 ± 0.93	99.46 ± 0.95	98.50 ± 2.33	.238

Mean ± SD, *p < 0.05; TMT, trail making task; rTMT, reversed trail making task.

### Group analysis of HbO, HbR, and HbT values

**Figure 3:**
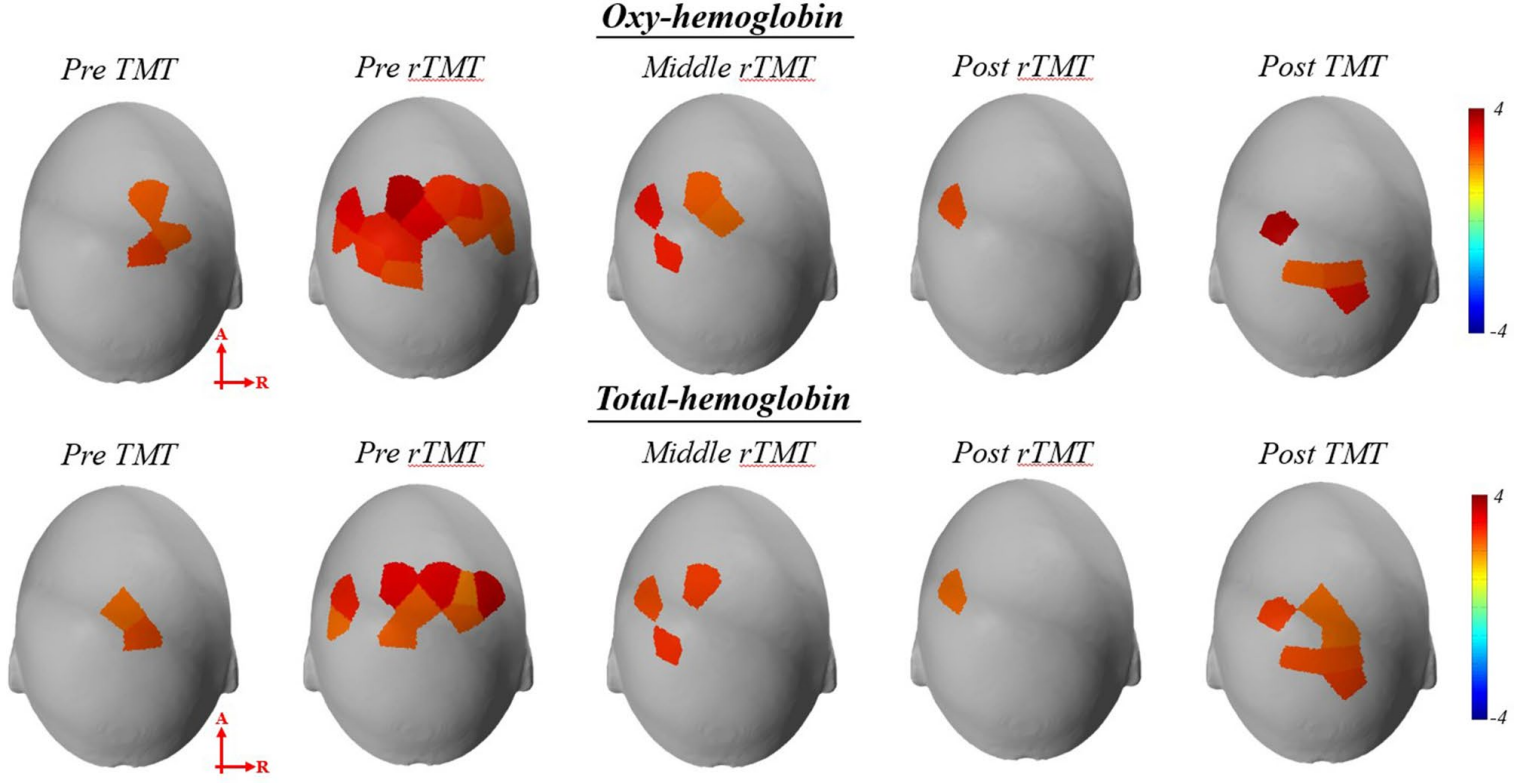
Group-average t-statistic maps of oxyhemoglobin (HbO) and total hemoglobin (HbT) values during trail making task and reversed trail making task using NIRS-Lab software (*p*_*uncorrected*_ < 0.05). TMT, trail making task; rTMT, reversed trail making task. Redder color represents more activation in each task compared to resing, vice versa.

In the group analysis, during the pre-TMT, the HbO values showed significant activation in the right SAC (*p*_*uncorrected*_ < 0.05). The HbO values revealed significant activation in the left V3 and bilateral SAC, AG, and SMG during the pre-rTMT (*p*_*uncorrected*_ < 0.05). HbO values showed significant activation in the bilateral SAC and left SMG during the middle-rTMT but only in the left SMG during the post-rTMT (*p*_*uncorrected*_ < 0.05). There was a significant increase in HbO values in the bilateral SAC, left V3, and right V2 during post-TMT (*p*_*uncorrected*_ < 0.05) (Table 2 and Fig. 3).

**Table 2:** Significant channels for oxyhemoglobin during trail making task and reversed trail making task.

Session	Brain region	Channel	Brodamann area	t value	*p*_uncorrected_
Pre TMT	Rt somatosensory association cortex	11	5	2.34	0.024
		12	7	2.30	0.027
		41	7	2.51	0.016
Pre rTMT	Lt somatosensory association cortex	7	5	3.58	0.009
		8	7	2.76	0.008
		25	7	2.65	0.011
		40	7	2.76	0.008
	Lt third visual cortex	2	19	2.38	0.022
	Lt angular gyrus	24	39	2.68	0.010
	Lt supramarginal gyrus	6	40	3.00	0.005
	Rt somatosensory association cortex	11	5	2.73	0.009
		12	7	2.45	0.019
		39	7	3.07	0.004
	Rt angular gyrus	29	39	2.26	0.029
	Rt supramarginal gyrus	10	40	2.38	0.022
Middle rTMT	Lt somatosensory association cortex	7	5	2.29	0.027
		25	7	2.75	0.008
	Lt supramarginal gyrus	6	40	2.96	0.005
	Rt somatosensory association cortex	39	7	2.25	0.030
Post rTMT	Lt supramarginal gyrus	6	40	2.39	0.021
Post TMT	Lt somatosensory association cortex	8	7	3.53	0.001
	Lt third visual cortex	2	19	2.36	0.023
	Rt somatosensory association cortex	3	7	2.45	0.019
	Rt second visual cortex	5	18	2.96	0.005

The level of significance was set at *p* = 0.05 (uncorrected), TMT, trail making task; rTMT, reversed trail making task.

Group analysis of HbT values revealed significant activation in the right SAC during TMT and in the left AG, bilateral SAC, and bilateral SMG during the pre-rTMT (*p*_*uncorrected*_ < 0.05). HbT values showed significant activation in the left SAC and SMG during the middle-rTMT but only in the left SMG during the post-rTMT (*p*_*uncorrected*_ < 0.05). During the post-TMT period, there was a significant increase in HbT values in the bilateral SAC, right V2, and left V3 (*p*_*uncorrected*_ < 0.05) (Table 3 and Fig. 3).

**Table 3:** Significant channels for total hemoglobin during trail making task and reversed trail making task.

Session	Brain region	Channel	Brodamann area	t value	*p*_uncorrected_
Pre TMT	Rt somatosensory association cortex	39	7	2.14	0.038
		41	7	2.48	0.017
Pre rTMT	Lt somatosensory association cortex	7	5	3.21	0.003
		40	7	2.34	0.024
	Lt angular gyrus	24	39	2.23	0.031
	Lt supramarginal gyrus	6	40	2.76	0.008
	Rt somatosensory association cortex	11	5	3.22	0.003
		12	7	2.27	0.028
		39	7	2.32	0.025
	Rt supramarginal gyrus	10	40	3.31	0.002
Middle rTMT	Lt somatosensory association cortex	7	5	2.52	0.016
		25	7	2.74	0.009
	Lt supramarginal gyrus	6	40	2.38	0.022
Post rTMT	Lt supramarginal gyrus	6	40	2.19	0.034
Post TMT	Lt somatosensory association cortex	8	7	2.53	0.015
	Lt third visual cortex	2	19	2.39	0.022
	Rt somatosensory association cortex	3	7	2.47	0.018
		39	7	2.17	0.036
		41	7	2.17	0.036
	Rt second visual cortex	5	18	2.59	0.013

The level of significance was set at *p* = 0.05 (uncorrected), TMT, trail making task; rTMT, reversed trail making task.

In the group analysis of HbR values, there was no significant activation during each session (*p*_uncorrected_ < 0.05).

### Comparison of beta coefficient of HbO, HbR and HbT between sessions

**Figure 4:**
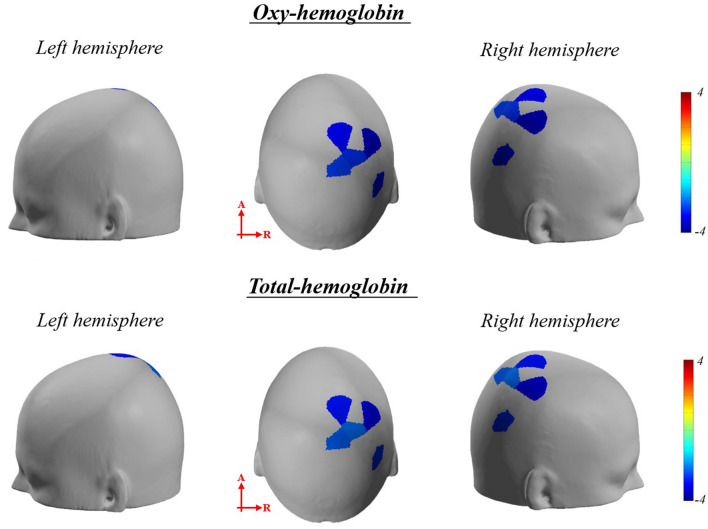
The significant channels of multiple comparisons of beta coefficient comparison between pre-rTMT and post-rTMT (contrast: pre-rTMT > post-rTMT). The p-value was corrected using the false discovery rate (*p*_corrected_ < 0.05). Bluer color represents lower activation in post-rTMT compared to pre-rTMT, vice versa.

**Figure 5:**
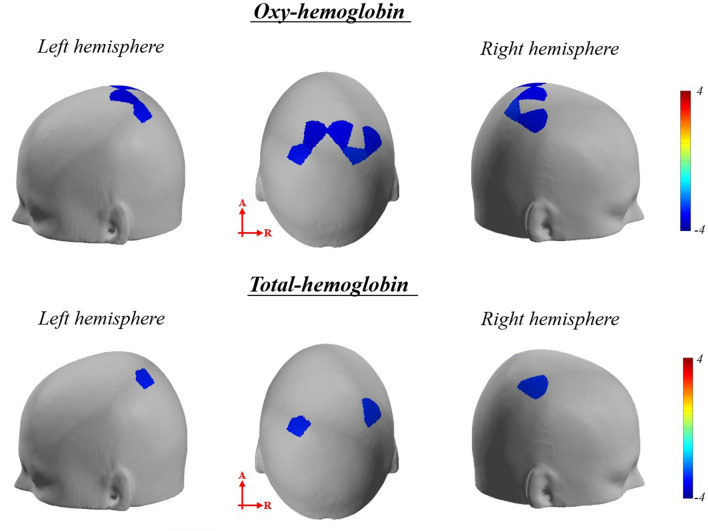
The significant channels of multiple comparisons of beta coefficient comparison between pre-TMT and post-TMT (contrast: pre-TMT > post-TMT). The p-value was corrected using the false discovery rate (*p*_corrected_ < 0.05). Bluer color represents lower activation in post-TMT compared to pre-TMT, vice versa.

The Friedman test of the beta coefficient of HbO revealed significant difference in the bilateral SAC, bilateral V1, bilateral SMG, left V2, left V3, and right AG between sessions (*p*_*corrected*_ < 0.05) (Supplementary Table S1). The post hoc analysis showed a significantly lower beta coefficient in the right SAC, AG, and SMG during pre-rTMT than during post-rTMT (*p*_*corrected*_ < 0.05) (Table 4 and Fig. 4). The beta coefficient in bilateral SAC and right SMG during post-TMT was significantly lower than during pre-TMT (*p*_*corrected*_ < 0.05) (Table 4 and Fig. 5).

**Table 4:** Significant channels of multiple comparisons of beta coefficient.

	Brain region	Channel	t value	*p*	*p*_corrected_
HbO					
Adaptation (pre-rTMT < post-rTMT)	Rt somatosensory association cortex (BA 5, 7)	11	− 3.111	0.002	0.01
		12	− 3.429	0.001	0.01
		41	− 3.727	< 0.001	< 0.001
	Rt angular gyrus (BA 39)	31	− 3.394	0.001	0.01
	Rt supramarginal gyrus (BA 40)	10	− 3.429	0.001	0.005
After effects (pre-TMT < post-TMT)	Lt somatosensory association cortex (BA 5,7)	7	− 2.683	0.007	0.035
		8	− 2.535	0.011	0.027
	Rt somatosensory association cortex (BA 5, 7)	11	− 2.828	0.005	0.017
		12	− 2.385	0.017	0.043
	Rt supramarginal gyrus (BA 40)	10	− 2.534	0.011	0.028
HbT					
Adaptation (pre-rTMT < post-rTMT)	Rt somatosensory association cortex (BA 5, 7)	11	− 3.111	0.002	0.01
		12	− 2.832	0.005	0.025
		41	− 3.876	< 0.001	0.005
	Rt angular gyrus (BA 39)	31	− 3.536	< 0.001	< 0.001
	Rt supramarginal gyrus (BA 40)	10	− 2.981	0.003	0.03
After effects (pre-TMT < post-TMT)	Lt somatosensory association cortex (BA 5,7)	8	− 2.830	0.012	0.042
	Rt supramarginal gyrus (BA 40)	10	− 2.740	0.014	0.048

The level of significance was set at *p* = 0.05 (ucorrected), TMT, trail making task; rTMT, reversed trail making task; BA, Broadmann area; The *p*-value was corrected using the false discovery rate (FDR).

The Friedman test of the beta coefficient of HbT revealed significant differences in bilateral SAC, bilateral AG, bilateral SMG, left V2, left V3, and right V1 between sessions (*p*_*corrected*_ < 0.05) (Supplementary Table S1). Post hoc analysis showed that the beta coefficients in the right SAC, AG, and SMG were significantly lower during post-rTMT than during pre-rTMT (*p*_*corrected*_ < 0.05) (Table 4 and Fig. 4). The beta coefficient in the left SAC and right SMG were significantly lower during post-TMT than during pre-TMT (*p*_*corrected*_ < 0.05) (Table 4 and Fig. 5).

In the Friedman test of the beta coefficient of HbR, there was no significant difference between sessions (*p*_corrected_ > 0.05).

## Discussion

The purpose of this study was to investigate the changes in performance and cortical activity based on hemodynamic response during sensorimotor adaptation to a reversed spatial orientation task in healthy adults using fNIRS. Four major findings were confirmed. First, the group analysis of HbO and HbT showed activation in the SAC during pre-TMT, pre-rTMT, middle-rTMT, and post-TMT. Functional neuroimaging studies demonstrated that the SAC is associated with visuospatial processing during visually guided reaching^38, 39^. In addition, this region is responsible for establishing visuomotor relationships through sensorimotor integration^38^. In the spatial orientation task, the construction of visuomotor mapping based on visuospatial processing and visuomotor integration was important to successfully connect targets. Therefore, the SAC plays a role in forming visuomotor relationships during TMT and rTMT.

Second, based on the group analysis of HbO and HbT, we observed sustained activation in the left SMG during pre-, middle-, and post-rTMT. These results were consistent with previous adaptation studies. In 2008, Girgenrath et al.^40^ investigated the brain regions involved in sensorimotor adaptation to visual and velocity-dependent distortion. They reported activation in bilateral SMG during the velocity dependent-distortion, while it was limited to the left SMG during the visual-dependent distortion. The SMG was associated with motor attention and reprogramming, which are component sensorimotor adaptation^41, 42^. In particular, left SMG connects the perception and motor execution by integrating the spatial and temporal variations^43^. Therefore, the persistence of activity in the left SMG is related to the motor attention and integration of perceptual spatiotemporal information, leading to sensorimotor adaptation to rTMT.

The third finding is that the performance of the spatial orientation task and the efficiency of cortical activity improved after rTMT training. To confirm whether sensorimotor adaptation had occurred, we compared the performance of the spatial orientation task between pre- and post-rTMT. As a result, response time and completion number improved after rTMT training. These results indicated that individuals adapted to rTMT, resulting in more and faster connections in a limited time of 40 s. In addition, we compared the beta coefficient of HbO and HbT between pre-rTMT and post-rTMT to investigate changes in cortical activity during sensorimotor adaptation. The beta coefficient of HbO and HbT in the SAC, AG, and SMG decreased after the rTMT training. Adaptation to rTMT required eye-hand coordination by integrating the discrepancy between visual feedback and motor output^12^. Previous studies suggested that the posterior parietal cortex is important for sensorimotor integration during adaptation^12, 44^. The posterior parietal cortex, through its connection with the primary sensory cortex, basal ganglia, and cerebellum, receives multisensory input from the primary sensory cortex and output from the cerebellum to facilitate sensorimotor integration^12, 44^. In addition, the multisensory integration leads to encoding spatial location for movement^45^, and ultimately develops a reference map of spatial orientation and navigation as well as a new sensorimotor transformation^46^. The efficiency of cortical activity improved when sensorimotor adaptation occurred^34, 47^. The fMRI studies demonstrated that cortical activity in the bilateral posterior parietal cortex decreased following visuomotor adaptation^48^. They suggested that the posterior parietal cortex is associated with visual attention, eye movements, and visuo-motor control. Similarly, we observed decreased cortical activity in the right posterior parietal cortex after sensorimotor adaptation of rTMT. This would be related to the contribution of the right posterior parietal cortex in establishing a visuomotor-transformation in the early stages of adaptation^49^. Therefore, our findings indicated that the efficiency of cortical activity in the right posterior parietal cortex and performances improved through sensorimotor adaptation.

The fourth major finding related to the after-effects. In general, the presence of after-effects in adaptation tasks is considered evidence of the formation of an internal model which is associated with implicit sensorimotor adaptation. We examined the presence of after-effects by comparing the performance of the spatial orientation task between pre- and post-TMT. Although there were no significant differences in average response time, completion number, error, and accuracy, we confirmed the increased response time at the early stage of post-TMT. The after-effect dissipates quickly as individuals rapidly adapt to the normal sensorimotor environment. It is referred to as the wash-out phase and appears quickly after approximately 50 trials in healthy adults when visual feedback is provided^6^. Wash-out reflects a recalibration to the original sensorimotor transformation as the new learned sensorimotor relationship is faded from memory^50^. Based on the previous studies, our results would be associated that individuals quickly revert and retrieve the original sensorimotor relationship.

We failed to detect activation based on the HbR in group analysis and the comparison of beta coefficients between sessions. The canonical HRF was applied to GLM analysis for HbO and HbR in the preset study. However, it is possible that this approach does not reflect the differences in temporal characteristics between HbO and HbR. Previous studies reported that the peak latency of the HbR was delayed compared to that of the HbO^51, 52^. Considering these differences in hemodynamic responses, it may be inappropriate to apply the same canonical HRF as a regressor for both hemoglobin parameters^53^. In addition, Uga et al.^54^ suggested that the adaptive HRF approach, considering the temporal characteristics of HbR, can increase the statistical power of HbR. Therefore, further studies should apply adaptive HRF in order to increase the statistical power of HbR.

## Conclusion

We demonstrated that efficiency of neural activation and performance of spatial orientation improved when adapting to a reversed spatial orientation task. These results provide an understanding of the neural mechanisms of sensorimotor adaptation. However, this study had several limitations. First, it is difficult to generalize the results of the current study to other age groups because our study participants were healthy adults in their 20 s; additionally, our study had a small sample size. Second, owing to methodological limitations, the present study did not use short-distance channels, which is a promising method to correct the fNIRS signal^47, 48^. Further studies should apply short distance channels in order to improve the quality of fNIRS signal.

### Supplementary Information

Supplementary Table S1.

## Data Availability

The datasets used and/or analyzed during the current study available from the corresponding author on reasonable request.
