# Supplementary material for: Sensorimotor adaptation in spatial orientation task: a fNIRS study
Source: Sci Rep. 2023 Sep 13;13:15160. doi: 10.1038/s41598-023-42416-3 (PMC10499899; doi:10.1038/s41598-023-42416-3)
Supplement: Supplementary file 1 — Supplementary Table S1. [file 41598_2023_42416_MOESM1_ESM.docx]

| Brain region | Channel | F | p | p_adj_ |
| --- | --- | --- | --- | --- |
| **OxyHb** |  |  |  |  |
| Lt somatosensory association cortex (BA 5, 7) | 7 | 12.356 | 0.015 | 0.036 |
|  | 8 | 17.943 | 0.001 | 0.010 |
|  | 25 | 15.733 | 0.003 | 0.015 |
| Lt primary visual cortex (BA 17) | 1 | 15.200 | 0.004 | 0.018 |
| Lt second visual cortex (BA 18) | 4 | 12.960 | 0.011 | 0.032 |
| Lt third visual cortex (BA 19) | 2 | 12.700 | 0.013 | 0.035 |
|  | 27 | 14.480 | 0.006 | 0.022 |
| Lt supramarginal gyrus (BA 40) | 6 | 11.289 | 0.024 | 0.049 |
|  | 41 | 20.711 | <0.001 | <0.001 |
| Rt somatosensory association cortex (BA 5, 7) | 3 | 12.356 | 0.015 | 0.038 |
|  | 11 | 18.240 | 0.001 | 0.006 |
|  | 12 | 17.511 | 0.002 | 0.011 |
|  | 30 | 20.089 | <0.001 | <0.001 |
|  | 39 | 14.400 | 0.006 | 0.019 |
| Rt primary visual cortex (BA 17) | 19 | 15.100 | 0.004 | 0.016 |
| Rt angular gyrus (BA 39) | 29 | 11.600 | 0.021 | 0.045 |
|  | 31 | 14.640 | 0.006 | 0.020 |
| Rt supramarginal gyrus (BA 40) | 10 | 18.578 | 0.001 | 0.082 |
|  | 34 | 11.840 | 0.019 | 0.043 |
| **HbT** |  |  |  |  |
| Lt somatosensory association cortex (BA 5, 7) | 8 | 12.914 | 0.012 | 0.037 |
|  | 25 | 14.311 | 0.006 | 0.024 |
| Lt second visual cortex (BA 18) | 4 | 14.800 | 0.005 | 0.029 |
| Lt third visual cortex (BA 19) | 2 | 11.800 | 0.019 | 0.043 |
|  | 27 | 15.280 | 0.004 | 0001 |
| Lt angular gyrus (BA 39) | 24 | 11.733 | 0.019 | 0.041 |
|  | 26 | 11.920 | 0.018 | 0.043 |
| Lt supramarginal gyrus (BA 40) | 6 | 14.222 | 0.007 | 0.026 |
|  | 41 | 18.756 | 0.001 | 0.020 |
| Rt somatosensory association cortex (BA 5, 7) | 3 | 18.222 | 0.001 | 0.041 |
|  | 11 | 17.040 | 0.002 | 0.027 |
|  | 12 | 14.044 | 0.007 | 0.023 |
|  | 30 | 15.022 | 0.005 | 0.025 |
|  | 39 | 12.356 | 0.015 | 0.041 |
| Rt primary visual cortex (BA 17) | 19 | 15.700 | 0.003 | 0.030 |
| Rt angular gyrus (BA 39) | 29 | 11.800 | 0.019 | 0.038 |
|  | 31 | 15.840 | 0.003 | 0.024 |
| Rt supramarginal gyrus (BA 40) | 10 | 14.489 | 0.006 | 0.027 |
|  | 34 | 11.680 | 0.02 | 0.037 |

**Supplementary Table 1.** Significant channels in the comparison of β coefficient of oxyHb and HbT between sessions.
